# Microbial Community Succession and Metabolite Changes During Fermentation of BS Sufu, the Fermented Black Soybean Curd by *Rhizopus microsporus*, *Rhizopus oryzae*, and *Actinomucor elegans*

**DOI:** 10.3389/fmicb.2021.665826

**Published:** 2021-06-25

**Authors:** Di Yao, Lei Xu, Mengna Wu, Xiaoyu Wang, Kun Wang, Zhijiang Li, Dongjie Zhang

**Affiliations:** ^1^College of Food Science and Engineering, Heilongjiang Bayi Agricultural University, Daqing, China; ^2^Heilongjiang Engineering Research Center for Coarse Cereals Processing and Quality Safety, Daqing, China; ^3^Key Laboratory of Agro-Products Processing and Quality Safety of Heilongjiang Province, Daqing, China; ^4^National Coarse Cereals Engineering Research Center, Heilongjiang Bayi Agricultural University, Daqing, China

**Keywords:** BS Sufu, microbial community succession, metabolite, high-throughput DNA sequencing, volatile flavor component

## Abstract

BS Sufu is a fermented food that is made by mixed black soybeans and soybeans. Microbial communities and metabolites play an important role for the final product. We characterized microbial diversity of BS Sufu during fermentation by high-throughput DNA sequencing. Meanwhile, volatile compounds were investigated by solid-phase microextraction (SPME) coupled with gas chromatography–mass spectrometry (GC-MS). The results showed that bacterial diversity was higher than that of fungi in BS Sufu. We found the existence of bacterial and fungal core communities, including *Enterococcus*, *Enterobacter*, *Rhizopus*, and *Monascus*. Network analysis indicated that bacterial and fungal communities maintain positive and negative interactions, which are important to shape the resident microbial communities in Sufu. In addition, 17 free amino acids (FAAs) were detected at the post-fermentation stage, and umami amino acid mainly contributed to taste of BS Sufu. Furtherly, a total of 79 volatile constituents in BS Sufu, including nine alcohols, 31 esters, and four aldehydes, form synergistically the unique odor of Sufu. Additionally, the correlations between microbiota and metabolites were analyzed. Our results suggested that these microbial taxa and metabolites contribute to the taste and flavor of BS Sufu. This study provided information for analysis of BS Sufu at different fermentation periods in terms of the microbial diversity and metabolites, and this information was important to understand the properties of mixed soybeans Sufu.

## Introduction

Sufu is a traditional fermented soybean food in China that shares similar shapes and fermentation mechanisms with cheese, but the taste and flavor are different (Wan H. et al., 2020). Sufu is normally consumed as a flavor enhancer and appetizer owing to its characteristic flavor, pleasant taste, and nutritional value in China and other Asian countries (Xie et al., 2018). Based on the strain used as a starter, Sufu can be classified into three different types, including mold-fermented Sufu, bacteria-fermented Sufu, and naturally fermented Sufu (Feng et al., 2013). The nutrition and taste of Sufu are affected by many factors, for example, the type of raw materials and the production process. Current studies on Sufu mainly focus on optimization of technological conditions, determination of flavor components, and microbial analysis during fermentation process, but there are few studies on the production of Sufu with various beans as raw materials. The content of protein and essential amino acids (EAAs) in black beans is higher than that of soybeans, and the content of phenolic substances is very rich (Meng et al., 2020). Therefore, we decided to use mixed black beans and soybeans (BS) as raw materials to make Sufu by processing technology of mold-fermented Sufu with minor modifications, which can promote nutrition diversification of traditional Sufu.

Sufu is produced under open or semi-open conditions via the actions of various microbes, including starter and indigenous microorganisms, which play important roles in flavor generation (Huang et al., 2018). At present, high-throughput sequencing (HTS) provides a reliable method to comprehensively describe the dynamics of microbial communities, and these studies have expanded our knowledge of the microbial community structure in Sufu. Recent published data indicate that Sufu microbiota showed a prevalence of bacteria genus belonging to *Lactococcus*, *Streptococcus*, *Enterobacter*, and *Acinetobacter* (Wan H. F. et al., 2020); and fungi such as *Trichosporon* and *Aspergillus* were also described. In addition, microbial abundances are mostly correlated with chemical characteristics, such as salinity, ethanol content, and environmental factor. Moreover, metabolomic approaches, such as gas chromatography (GC), mass spectrometry (MS), and high-performance liquid chromatography (HPLC) have been applied to determine the metabolite profiles of Sufu (Moy and Chou, 2010). However, few systematic studies have evaluated the correlation on the microbial compositions and metabolites of Sufu at different fermentation stages; so far, there is no study on the characteristics of mixed beans Sufu. Accordingly, in this study, we aimed to explore the diversity and composition of the microbial community, the profile of metabolites, including amino acids and flavor compounds, and the correlation between microbes and metabolites in BS Sufu. The results will help us to improve understanding of the role of microorganisms in the production of Sufu, which will provide a certain reference for research and development of a new type of Sufu.

## Materials and Methods

### BS Sufu Preparation and Sample Collection

The isolated strains *Rhizopus microsporus* SE-3 and *Rhizopus oryzae* CD-1 (derived from spontaneous fermented Sufu in our previous study) combined with *Actinomucor elegans* CCIC3093 (China Center of Industrial Culture Collection) of 1:1:1 ratio were used as the starter for inoculation and fermentation. Black beans (Jihei 6) and soybeans (Heihe 43) (Jiansanjiang, Heilongjiang, China) of 1:1 ratio were used as raw materials to prepare Sufu, which is called BS Sufu. Simply, pehtze is prepared by inoculating mixed strain on the surface of tofu cubes, then the pehtze is salted for 24 h, and the salted pehtze is dispensed into wide-mouthed bottles and ripened in the dressing mixture for 60 days. The dressing mixture of BS Sufu mainly consists of food-grade monascus pigment, yellow rice wine, high-concentration liquor, and other spices (Supplementary Figure 1). Sufu samples were collected periodically at different stages of fermentation: pehtze (BS_M), salted pehtze (BS_Y), and post-fermentation 10 days (BS_10), 30 days (BS_30), and 60 days (BS_60). The samples were collected and stored at −80°C.

### Free Amino Acid Detection of BS Sufu

Free amino acids (FAAs) were detected using Amino Acid Analyzer (Model L-8900, Hitachi, Tokyo, Japan). The sample was extracted with 0.01 mol/L of HCl ultrasonically for 5 min and centrifuged at 4,000 rpm for 10 min; then the supernatant was added with 2%∼4% sulfosalicylic acid (Solarbio, Beijing, China), centrifuged, and filtered by a 0.22-μm filter for measurement. The chromatographic conditions were as follows: 2622#PH ion exchange chromatography column (4.5 × 60 mm), column temperature (57°C), flow rate (0.4 ml/min), and injection volume (20 μl).

### Volatile Flavor Composition Analysis of BS Sufu Through Headspace Solid-Phase Microextraction–Gas Chromatography–Mass Spectrometry

Individual samples (5 g) were mashed and placed into 20-ml headspace vial. Then, the vial was sealed after adding isoamyl phenylacetate (Solarbio, Beijing, China) and heated in water bath at 60°C for 20 min. After that, the solid-phase microextraction needle (57348-U, Supelco, Bellefonte, Pennsylvania, United States) was inserted into the vial to extract for 30 min (the needle being aged at 250°C before use) and immediately inserted into the GC inlet, followed by 3 min of desorption. Subsequently, the GC-MS analysis of volatile compounds was performed on Agilent 7890A-5975C GC-MS equipped with HP-5MS chromatographic column (60 m × 250 μm × 0.25 μm). High-purity helium (purity > 99.999%) was used as the carrier gas at a constant flow of 1.5 ml/min, and the inlet temperature was set at 250°C. Oven starting temperature was 50°C, programmed at 5°C/min to 100°C for 2 min, programmed at 4°C/min to 140°C for 1 min, programmed at 4°C/min to 180°C for 2 min, and finally programmed to 250°C at 5°C/min and held at 250°C for 5 min. The conditions of MS were ion source temperature 230°C, quadrupole temperature 150°C, electron ionization (EI) 70 eV, and full scan 35∼550 da.

### DNA Extraction and High-Throughput Sequencing

Total genomic DNA was extracted using an Ezup Column Genomic DNA Purification Kit (Sangon Biotech, Shanghai, China), according to the manufacturer’s protocol. The quality and quantity of the extracted DNA were assessed by the ratios of 260 nm/280 nm and 260 nm/230 nm with a NanoDrop spectrophotometer (A360, AOE, Shanghai, China). The V3–V4 region of the bacterial 16S rRNA gene and ITS1 region of the fungi were amplified using TransStart Fastpfu DNA Polymerase (TransGen, Beijing, China). Then PCR products were purified using the SanPerp Column PCR Product Purification Kit (Sangon Biotech, Shanghai, China). The 16S rRNA and ITS1 gene amplicons were sequenced using Illumina HiSeq deep sequencing (Majorbio, Shanghai, China).

### Bioinformatics Analysis

The double-end sequence data obtained by HiSeq sequencing were spliced or merged into a sequence tag, and quality control filtering was performed on the quality of reads and the effect of the merging. Then, the stitching sequence obtained is the original tag data. The read sequences were aligned with species annotation databases through the UCHIME Algorithm. Then, the chimera sequence was identified and removed to obtain the final valid data. Usearch software was used to cluster the tags at a similarity level of 97% to obtain operational taxonomic units (OTUs) (Hang et al., 2014). Alpha diversity was evaluated using the Mothur v.1.11.0 program, and Kyoto Encyclopedia of Genes and Genomes (KEGG) function of 16S RNA gene was annotated by Tax4Fun and Silva database. Ultimately, redundancy analysis (RDA) was performed using Canoco software to reflect the relationship between the core microorganisms and flavor compounds (Barka, 2019).

### Statistics Analysis

SPSS 22.0 was used to calculate the significance of difference by ANOVA; the results were expressed as the mean ± SD.

### Sequence Accession

The datasets (raw sequences) generated and analyzed during this study have been submitted to National Center for Biotechnology Information (NCBI) Sequence Read Archive (SRA) repository with accession number PRJNA693985 and can be accessed through the following link: http://www.ncbi.nlm.nih.gov/sra/PRJNA693985.

## Results

### Free Amino Acid Composition and Content of BS Sufu

Seventeen FAAs were detected in BS Sufu at the post-fermentation stage (Table 1). The content of all FAAs reached the highest in BS_60, except for ARG. FAAs have the characteristics of low taste threshold and strong taste ability. According to the taste characteristics of FAAs, they are grouped into three categories, including umami amino acids (UAAs), sweet amino acids (SAAs), and bitter amino acids (BAAs). The content of total SAAs and UAAs accounted for 62.5%, and total BAAs accounted for 37.5%. The content of seven EAAs was relatively stable, maintaining about 37% in post-fermentation stages. The taste intensity is affected by both the content of the taste substance and the threshold value. TAV is the ratio between the concentration of the taste substance and its threshold value. The value (TAV > 1) can be considered as the food taste contribution: the greater the TAV value, the greater the contribution of FAAs to taste. In the BS_10 stage, FAAs with TAV > 1 included ASP, GLU, ALA, VAL, and HIS; FAAs in the BS_30 stage includes ASP, GLU, PHE, ALA, VAL, LYS, and HIS; the BS_60 stage includes ASP, GLU, PHE, ALA, VAL, MET, ILE, TYR, LYS, and HIS. In total, the kinds of BAAs were significantly higher than those of UAAs and SAAs, but the TAV value of UAAs was significantly higher than that of other FAAs, indicating that UAAs contribute to the taste of BS Sufu.

**TABLE 1:** Free amino acid content and TAV value of BS Sufu during post-fermentation.

**Taste**	**FAA**	**Threshold (mg/100g)**	**BS_10**		**BS_30**		**BS_60**	
			**content (mg/100g)**	**TVA**	**Content (mg/100g)**	**TVA**	**Content (mg/100g)**	**TVA**
	ASP	3	71.5 ± 0.13^a^	23.8	96.6 ± 0.57^b^	32.2	164.6 ± 0.94^c^	54.9
	PHE*	90	76.5 ± 0.35^a^	0.85	103.9 ± 0.12^b^	1.15	123.9 ± 1.33^c^	1.38
	GLU	5	256.6 ± 1.39^a^	51.3	371.8 ± 1.44^b^	74.4	500.9 ± 1.17^c^	100.2
TUAA			404.6^a^		572.3^b^		789.3^c^	
	THR*	260	39.2 ± 2.27^a^	0.15	56.9 ± 3.25^b^	0.22	80.0 ± 1.19^c^	0.31
	SER	150	44.2 ± 1.52^a^	0.29	66.8 ± 0.31^b^	0.44	82.8 ± 4.51^c^	0.55
	PRO	300	40.9 ± 0.12^a^	0.14	48.4 ± 0.02^b^	0.16	79.9 ± 0.61^c^	0.27
	GLY	130	33.7 ± 1.47^a^	0.26	45.8 ± 1.92^b^	0.35	67.1 ± 0.48^c^	0.52
	ALA	60	86.8 ± 3.56^a^	1.44	116.0 ± 2.35^b^	1.93	129.9 ± 1.43^c^	2.16
TSAA			244.8^a^		333.9^b^		439.7^c^	
	CYS	/	8.5 ± 0.04^a^	/	14.3 ± 0.47^b^	/	20.2 ± 0.077^c^	
	VAL*	40	63.7 ± 2.76^a^	1.59	87.3 ± 0.03^b^	2.18	112.1 ± 0.52^c^	2.81
	MET*	30	16.7 ± 0.42^a^	0.56	21.7 ± 0.02^b^	0.72	31.2 ± 0.08^c^	1.04
	ILE*	90	55.7 ± 0.69^a^	0.62	77.9 ± 0.46^b^	0.86	105.9 ± 1.34^c^	1.18
	LEU*	190	90.4 ± 1.87^a^	0.48	126.7 ± 1.63^b^	0.66	163.1 ± 1.59^c^	0.85
	TYR	91	61.1 ± 0.73^a^	0.67	88.3 ± 0.95^b^	0.97	109.0 ± 0.84^c^	1.20
	LYS*	50	44.2 ± 0.01^a^	0.88	69.3 ± 0.07^b^	1.38	109.4 ± 0.55^c^	2.19
	HIS	20	52.9 ± 0.33^a^	2.64	72.0 ± 0.34^b^	3.60	83.2 ± 1.67^c^	4.16
	ARG	50	2.6 ± 0.01^b^	0.05	2.4 ± 0.02^a^	0.05	2.5 ± 0.04^b^	0.05
TBAA			395.8^a^		559.9^b^		736.6^c^	
TFAA			1045.2^a^		1466.9^b^		1965.6^c^	
EAA			386.4^a^		543.7^b^		725.6^c^	
EAA/TFAA			36.9%		37.1%		36.9%	

Different letters indicate the significant difference (*P* < 0.05) of BS Sufu at different fermentation times; Threshold is the lowest taste concentration of amino acids; TVA, amino acid content/amino acid taste threshold; TFAA, total free amino acids; TUAA, total umami amino acid; TSAA, total sweet amino acid; TBAA, total bitter amino acid; EAA (*), essential amino acid.

### Volatile Flavor Composition Analysis of BS Sufu

A total of 79 volatile constituents in BS Sufu at the post-fermentation stage, including nine alcohols, 31 esters, four aldehydes, seven ketones, two acids, eight olefins, seven alkanes, three ethers, and eight other organic compounds, form synergistically the unique odor of Sufu (Table 2). These volatile compounds in three fermentation stages were different. Compared with BS_30, BS_60 had fewer kinds of flavor compounds, but the content was higher. For example, in the BS_60 stage, the total amount of alcohols and esters compounds was significantly higher than that of the BS_30 stage. Esters accounted for the highest proportion in all compounds identified of BS Sufu, which mainly contribute to the complex flavor of BS Sufu. The relative content of ethyl caproate, isoamyl phenylacetate, ethyl palmitate, ethyl linoleate, and ethyl alcohol was higher than that of other flavor compounds. The relative content of ethyl alcohol and phenethyl alcohol was higher among the alcohol components. In addition, the aldehyde, ketone, alkane, and olefin compounds were also identified, but the content was extremely low, which contributes less to the flavor of BS Sufu.

**TABLE 2:** Identification and relative content of volatile components in BS Sufu.

**R.T./min**	**Category**	**Components**	**Relative content /%**			**CAS**
			**BS_10**	**BS_30**	**BS_60**	
4.1867	Alcohols	2-Pentanol	/	/	14.7885	006032-29-7
4.1514		Ethyl alcohol	19.5085	16.8737	6.7793	000064-17-5
6.5336		3-methyl-Pentanediol	/	/	2.6265	004457-71-0
6.5572		3-Isopentanol	2.1987	1.9603	/	000123-51-3
14.6978		2-Ethylhexanol	0.1544	0.0961	0.1522	000104-76-7
17.4388		Linalool	0.0705	0.0899	/	000078-70-6
18.1153		Phenethyl alcohol	5.7259	4.5261	3.9577	000060-12-8
20.621		4-terpineol	0.0856	0.1203	0.115	000562-74-3
20.7327		2,3-dimethyl-3-Buten-2-ol	/	0.0478	/	010473-13-9
		Total alcohols	27.7436	23.7142	28.4192	
7.8747	Esters	Ethyl butyrate	0.2682	0.3002	0.8592	000105-54-4
9.0863		2-methyl-butanoic acid-ethyl ester	/	/	0.1727	007452-79-1
11.5451		N-(trifluoroacetyl)Glycine 1-methylbutyl ester	0.0619	/	/	057983-43-4
13.5509		Ethyl caproate	5.0352	6.9071	8.2835	000123-66-0
15.1978		Hexanoic acid, 2- ethyl-, methyl ester	0.0863	/	/	000816-19-3
15.8272		Ethyl dl-2-hydroxycaproate	0.0686	0.0738	/	006946-90-3
15.1977		2-ethyl-hexanoic acid-methyl ester	/	/	0.0969	000816-19-3
17.2859		Ethyl heptanoate	0.1737	0.2177	0.2454	000106-30-9
17.7565		2-ethyl caproate	0.0341	0.0407	0.1763	002983-37-1
20.2975		Ethyl benzoate	0.0362	0.0601	0.0893	000093-89-0
20.4327		Diethyl succinate	0.1437	0.0991	0.0662	000123-25-1
20.5151		Methyl phenylacetate	0.3262	0.3143	0.3161	000101-41-7
21.1033		Ethyl octanoate	8.6792	10.5167	11.5905	000106-32-1
21.9679		Ethyl nicotinate	0.0524	0.0165	/	000614-18-6
22.9384		2-Ethyl octenoate	/	/	0.0173	007367-82-0
23.009		Ethyl phenylacetate	0.1337	0.2193	0.1892	000101-97-3
23.1561		2-methylbutyl hexanoate	0.0402	0.0415	0.0422	002601-13-0
24.6912		Ethyl nonanoate	/	/	0.4514	000123-29-5
26.5029		Iso butyl octanoate	0.0475	0.0613	0.0329	000589-75-3
28.1146		Ethyl decanoate	0.5503	1.0277	0.7137	000110-38-3
29.7968		Isoamyl octanoate	0.0408	0.0664	0.0329	002035-99-6
30.1733		Ethyl octanoate	0.0942	0.0781	/	000106-32-1
31.4614		Isoamyl phenylacetate	16.1943	6.0555	17.7417	000102-19-2
34.4201		Ethyl laurate	0.0628	0.1504	0.0766	000106-33-2
41.0255		Ethyl myristate	0.3619	0.5198	0.2331	000124-06-1
50.3425		Ethyl stearate	0.0375	0.0428	0.1708	000111-61-5
44.5488		Methyl palmitate	/	0.0536	/	000112-39-0
45.7076		Ethyl (Z)-hexadec-9-enoate	0.0887	0.1437	0.0487	056219-10-4
46.1781		Ethyl palmitate	6.5381	7.7176	3.9638	000628-97-7
49.7955		Ethyl linoleate	6.7321	7.7067	3.6817	000544-35-4
49.8838		Ethyl oleate	4.9317	5.7179	2.9331	000111-62-6
		Total esters	50.8195	48.1485	52.2252	
15.4625	Aldehydes	Phenylethanal	0.0716	0.1016	0.1106	000122-78-1
24.1208		2-phenyl-2-butenal	0.0781	0.1028	0.0351	004411-89-6
25.5677		*Trans-*2,4-decadienal	0.0519	/	/	025152-84-5
27.2499		Gamma-nonalactone	0.0288	0.0331	/	000104-61-0
		Total aldehydes	0.2304	0.2375	0.1457	
10.1628	Ketones	Heptan-2-one	0.1113	0.0784	0.3127	000110-43-0
17.1035		2-Nonanone	0.3547	0.489	0.5144	000821-55-6
23.509		Piperitone	0.2403	0.2605	0.1848	000089-81-6
9.6805		2-Trifluoromethyl-2-hydroxy-5,5-dimethyl-Oxan-4-one	/	/	0.158	212615-80-0
11.5509		1-(3-ethylcyclobutyl)ethanone	/	/	0.1276	056335-71-8
20.7327		Dihydro-5-pentyl-2(3H)-Furanone	/	/	0.0952	000104-61-0
25.4089		2,5-Octanedione	0.1457	0.122	/	003214-41-3
		Total ketones	0.852	0.9499	1.3927	
17.9329	Acids	Ethylhexanoic acid	0.1048	0.1201	0.1274	000149-57-5
20.0387		Octanoic acid	0.329	0.5957	0.2022	000124-07-2
		Total acids	0.4338	0.7158	0.3296	
14.8508	Olefins	DL-Limonene	1.1599	0.2963	0.4818	000138-86-3
15.9801		Gamma-terpinene	/	0.0405	/	000099-85-4
17.4388		3,7-Dimethyl-1,3,6-Octatriene	/	/	0.0799	013877-91-3
27.8617		Alpha-copaene	/	0.0198	/	003856-25-5
29.0557		Alpha-Bergamotene	/	0.0164	/	017699-05-7
29.4086		*Trans-*Caryophyllene	/	0.0143	/	000087-44-5
32.8554		Alpha-terpinene	/	0.051	/	000099-86-5
10.251		Styrene	/	0.0308		000100-42-5
		Total olefins	1.1599	0.4691	0.5617	
8.3276	Alkanes	Hexamethyl-cyclotrisiloxane	/	/	0.204	000541-05-9
9.6923		1-Fluoro-heptane	0.1206	/	/	000661-11-0
25.6971		Dodecamethyl-cyclohexasiloxane	0.3575	0.1257	0.1377	000540-97-6
19.3269		Decamethylcyclopentasiloxane	/	0.1664	0.1327	000541-02-6
36.9729		Hexadecamethyl-cyclooctasiloxane	0.0355	/	/	000556-68-3
28.6028		Tetradecamethyl-hexasiloxane	/	0.0254	0.0184	000107-52-8
41.9725		Octadecamethyl-cyclononasiloxane	/	0.0189	/	000556-71-8
		Total alkanes	0.5136	0.3364	0.4928	
20.815	Ethers	4-Octenoic acid, ethyl ether	/	/	0.0526	1000132-45-5
21.3621		4-Allylanisole	/	0.0973	0.0799	000140-67-0
29.691		3,4-Difluoroanisole	/	0.0614	/	115144-40-6
		Total ethers	0	0.1587	0.1325	
12.8568	Others	Phenol	8.076	/	8.9403	000108-95-2
13.3626		2-Pentylfuran	/	0.8224	1.1933	003777-69-3
16.6212		Methacrylamide	0.0339	/	/	000079-39-0
17.0095		2,3,5,6-Tetramethylpyrazine	0.0311	0.0199	0.0279	001124-11-4
24.5854		*Cis-*Anethole	4.4738	/	5.0071	000104-46-1
24.9089		Indole	1.209	0.6083	0.5918	000120-72-9
29.691		Isothiocyanatoacetonitrile	0.0203	/	/	032772-75-1
37.6375		Xanthoxylin	0.1029	/	/	000090-24-4
		Total others	13.947	1.4506	15.7604	

*/ means that the volatile substance is not detected.*

### Microbial Community Dynamics of BS Sufu During Fermentation

After sequencing and quality control, the clean reads of all samples were clustered, revealing 346 and 168 OTUs of bacteria and fungi at 97% similarity level, respectively. Furthermore, alpha diversity was analyzed (Table 3). The Shannon index of bacteria was generally higher than that of fungi (*P* < 0.05), which proved that bacterial diversity was higher than that of fungi in BS Sufu. At the end of fermentation, the Shannon index of bacteria and fungi were reduced. Likewise, abundance-based coverage estimator (ACE) index indicated that the abundance of bacterial species was significantly higher than that of fungi. The coverage of samples at different fermentation stages is greater than 99%, indicating that sequencing has a large coverage of species, and showed that the sequencing depth was sufficient, which proved the reliability of the sequencing results and could represent the true composition of microorganisms in BS Sufu.

**TABLE 3:** The microbial diversity index of BS Sufu.

**Sample**	**Shannon**		**Simpson**		**ACE**		**Chao**		**Coverage**	
	**Bacteria**	**Fungi**	**Bacteria**	**Fungi**	**Bacteria**	**Fungi**	**Bacteria**	**Fungi**	**Bacteria**	**Fungi**
BS_M	1.71±0.27^a^	1.12±0.42^b^	0.30±0.07^a^	0.51±0.11^b^	144.4±89.63^a^	26.74±5.27^b^	130.08±96.28^b^	26.33±4.61^a^	0.9998	0.9999
BS_Y	1.69±0.09^a^	0.54±0.09^b^	0.28±0.05^a^	0.75±0.06^b^	63.9±19.91^a^	26.9±18.02^b^	57.37±11.01^b^	18.86±8.82^a^	0.9997	0.9999
BS_10	1.45±0.37^a^	1.16±0.21^b^	0.39±0.15^a^	0.62±0.08^b^	66.39±21.38^a^	51.71±2.92^b^	58.78±10.65^a^	49.44±2.57^a^	0.9997	0.9999
BS_30	1.74±0.50^a^	0.96±0.88^b^	0.28±0.10^a^	0.68±0.31^b^	101.4±42.91^a^	41.73±15.3^b^	96.26±45.11^b^	40.83±15.61^a^	0.9998	0.9999
BS_60	1.09±0.28^a^	0.75±0.38^b^	0.49±0.18^a^	0.76±0.14^b^	67.48±14.97^a^	39.96±13.12^b^	62.47±6.33^b^	39±13.14^a^	0.9994	0.9999

*Different letters indicate the significant difference between bacteria and fungi in a certain fermentation stage (*P* < 0.05).*

**FIGURE 1:**
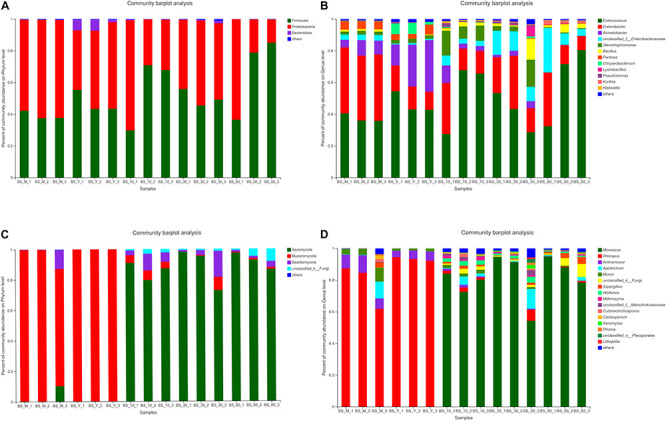
Species distribution and abundance of BS Sufu in different fermentation stages. The relative abundance of each taxon was defined as the percentage of the same taxon to the corresponding total sequences for each sample. Phylum [**(A)**, bacteria; **(C)**, fungi]; genus [**(B)**, bacteria; **(D)**, fungi]. Some classes with abundances of less than 1% were summarized as “other.”

Bacterial community dynamics were evaluated based on changes in relative abundance at phylum and genus levels (Figures 1A,B). Firmicutes, Proteobacteria, and Bacteroides were the main phyla, which exist in the whole fermentation process. At the genus level, the abundances of *Enterococcus* and *Enterobacter* were higher. At the stage of post-fermentation, the abundance of *Acinetobacter* decreased from 23.91% to 5.74%, while the abundance of *Enterobacter* and *Enterococcus* increased to 19.92% and 53.67%, respectively. During the whole post-fermentation process, the abundance of *Enterococcus* was the highest, the abundance of *Bacillus* increased, and *Acinetobacter* was almost undetectable.

The phylum of fungi included Ascomycota, Mucoromycota, and Basidiomycota (Figure 1C). The Mucoromycota was mainly concentrated in the pre-fermentation stage, but the Ascomycota and Basidiomycota were concentrated in the post-fermentation stage. The distribution of fungi genus is shown in Figure 1D. The abundance of *Rhizopus* in the pre-fermentation stage is 77.96% in the BS_M stage and 93.35% in the BS_Y stage, while the abundances of *Mucor* and *Actinomucor* declined in the post-fermentation stage. In general, the diversity of fungi elevated in the post-fermentation stage; for instance, *Wallemia*, *Millerozyma*, and *Xeromyces* begin to appear, and the abundance of *Monascus* was the highest in entire post-fermentation process. In summary, BS Sufu had significantly more bacterial species than fungi during the pre-fermentation stage, but the identified fungal species are slightly higher than bacteria during the post-fermentation stage.

### Comparison of Microbial Communities in BS Sufu at Different Fermentation Stages

The Lefse analysis was performed to uncover differentially abundant biomarkers by an Linear discriminant analysis (LDA) score (log 10) > 4 (Supplementary Figure 2). The *unclassified_Bacteria* was significantly enriched in BS_M, with an LDA value of 4.8. *Acinetobacter* (5.1) and *Chryseobacterium* (4.5) were significantly enriched in BS_Y, which contributed to the difference between BS_Y and other samples. Only the LDA score of *Pseudomonas* (3.4) at BS_10 was calculated, indicating a little contribution to the difference. *Stenotrophomonas* (4.7) was significantly enriched in BS_30; Enterobacteriaceae and *Kurthia* were significantly enriched in BS_60, demonstrating that their abundance was different from that of other species. Compared with bacteria, the LDA results of fungi were more unambiguous. *Actinomucor* (4.8) had a greater impact on the difference between BS_M and other samples. The significantly enriched species in BS_Y was *Rhizopus*, and significantly enriched species in BS_10 was *Wallemia*. Compared with other fermentation stages, there are obviously more enriched species in the BS_60 stage; nevertheless, only *Monascus* had a greater contribution to sample differences.

### Network Inference of Species in BS Sufu

**FIGURE 2:**
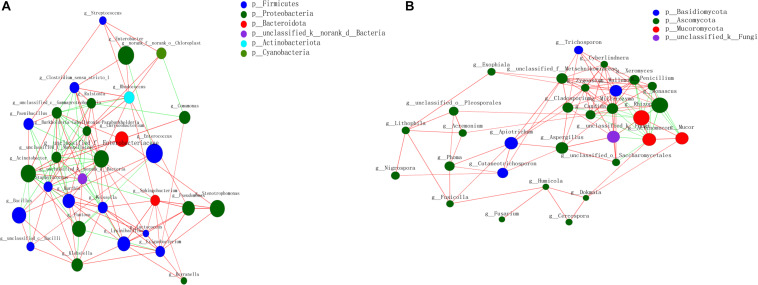
Horizontal correlation network diagram of BS Sufu bacteria **(A)** and fungi **(B)**. Color and size of the nodes represent relative abundance of different microbiota genus, respectively. Red indicates positive correlation, and green indicates negative correlation. The thickness of the line indicates the size of the correlation coefficient. p, phylum; g, genus.

The relationship between BS Sufu microorganisms based on the relevant network diagram was studied, and the correlation coefficient in different species was calculated (Figure 2). The correlation network diagram of genus level revealed that there were significant interactions between different genera of the BS microorganisms. In the bacterial network diagram, *Acinetobacter* and Enterobacteriaceae had a significantly positive correlation, and the clustering coefficient was 0.504. The number of genera connected to Enterobacteriaceae and *Staphylococcus* was the largest (degree = 12) (degree represents the number of one node connected to other node), followed by *Weissella*, but the clustering coefficients of *Weissella* were small (0.348), and *Pseudomonas* and *Lactococcus* had the largest clustering coefficients of 0.669. There was an obviously positive correlation between *Lactococcus* and other bacterial genera. The clustering coefficient indicated the importance of this genus in the entire microbial environment.

In the fungi network diagram, *Mucor* has the biggest positive correlation with *Actinomucor* and *Rhizopus* in the pre-fermentation stage. The number of fungi genera interconnected with *Millerozyma* was the largest (degree = 15). Interestingly, *Monascus* added in the post-fermentation process is positively correlated with *Rhizopus* and *Mucor*. In addition, *Zygosaccharomyces* had a significantly positive correlation with other genera.

### Correlation Analysis of Microbial Diversity and Metabolites of BS Sufu

**FIGURE 3:**
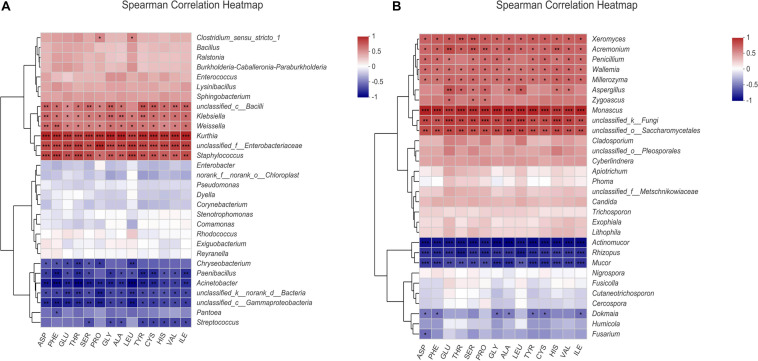
Heat map of the correlation between amino acids and microorganisms of BS Sufu: **(A)** bacteria and **(B)** fungi. The figure shows 14 free amino acids with high content; * represents the correlation between free amino acids and species (***P < 0.001, **P < 0.01, *P < 0.05). Colors closer to red mean stronger positive correlation; colors close to blue means stronger negative correlation.

Bacteria Pearson correlation heat map (Figure 3A) indicated that all FAAs were positively correlated with *Kurthia*, *unclassified_Enterobacteriaceae*, *Staphylococcus*, *Weissella*, and *Klebsiella*. Besides, all FAAs had obviously negative correlation with *Acinetobacter*, *Gammaproteobacteria*, *Paenibacillus*, and *Streptococcus*. The fungal correlation is shown in Figure 3B. All amino acids had an obviously positive correlation with *Monascus*, *unclassified_Saccharomycetales*, *Wallemia*, *Millerozyma*, and *Xeromyces*. Furthermore, *Aspergillus* and *Zygoascus* were also positively correlated with GLU, SER, PRO, etc.

**FIGURE 4:**
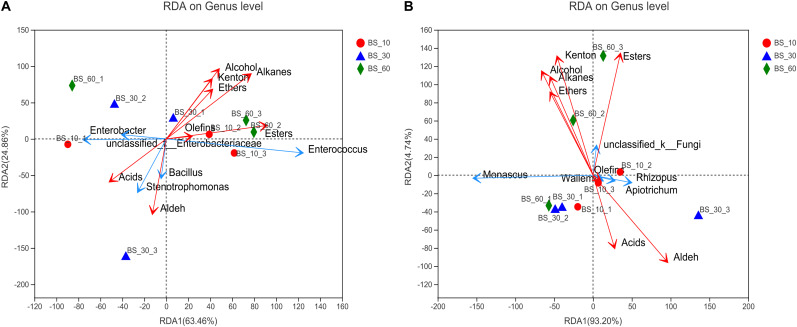
Redundancy analysis (RDA) of the correlation between flavor compounds and microorganisms of BS Sufu: **(A)** bacteria and **(B)** fungi. The blue arrow represents the species; the red arrow represents flavor compounds. The longer the arrow, the stronger the correlation.

Redundancy analysis revealed the correlation between dominant genus and flavor compounds (Figure 4). *Enterococcus* had a significantly positive correlation with esters, alcohols, ketones, alkanes, and ethers. *Stenotrophomonas* and *Bacillus* were significantly positively correlated with organic acids and aldehydes. *Enterobacter* and *unclassified-Enterobacteriaceae* were positively correlated with organic acids but negatively correlated with the other types of compounds. *Unclassified-fungi* had a significantly positive correlation with alcohols, esters, ketones, and ethers, which were concentrated in the end of post-fermentation. *Rhizopus* and *Apiotrichum* were positively related with organic acids and aldehydes, but the two substances were concentrated in the early stage of post-fermentation. *Monascus* was also positively correlated with alcohols, ethers, and ketones and negatively correlated with esters and organic acids.

### Functional Prediction of Bacteria Gene

**FIGURE 5:**
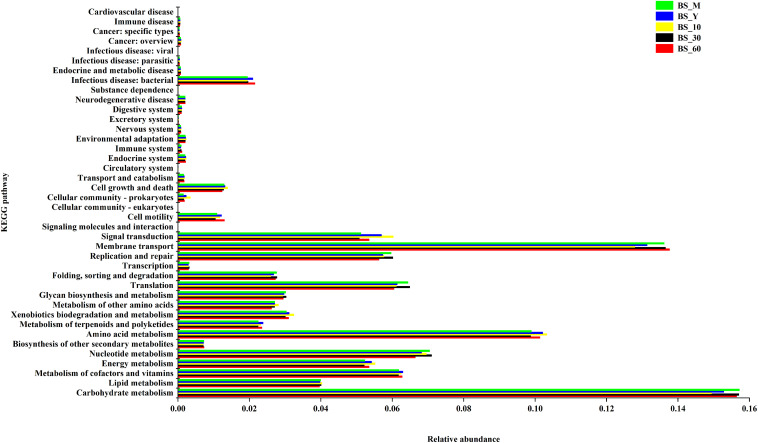
Kyoto Encyclopedia of Genes and Genomes (KEGG) function annotation of bacteria in BS Sufu.

As shown in Figure 5, the function of bacterial genes was mostly annotated in metabolism (59.9%), genetic information processing (15.1%), environmental information processing (18.9%), cellular processes (2.8%), human diseases (2.5%), and biological systems (0.7%). Among them, carbohydrate metabolism accounted for 25.8% of metabolism, lipids 6.7%, amino acids 21.4%, and metabolism of terpenoids and polyketides 3.9%. The carbohydrate metabolism was more abundant in BS_M, BS_30, and BS_60. The amino acid metabolism was enriched in BS_Y, BS_10, and BS_60. In general, the genes involved in carbohydrate metabolism were more than those in amino acid metabolism. The function prediction showed that majority of the functional information of bacterial community in BS Sufu was meaningful.

## Discussion

The fermentation process of Sufu is complicated and affected by various factors (Liu et al., 2018). As a fermented food rich in protein, protein degradation and amino acid metabolism are extremely key in the formation of flavor substances. The content of FAAs depends on the ratio of their generation and degradation (Tang et al., 2018). ARG has the function of anti-tumor and regulating immunity (Hui et al., 2017; Mondanelli et al., 2017). LYS can increase the absorption and accumulation of calcium, accelerate bone growth, enhance immunity, and promote development (Korompokis et al., 2016). PHE can synthesize important neurotransmitters and hormones to improve memory in the body (Kumar et al., 2017; Souza et al., 2017). LEU can improve the recovery ability of muscle fibers and make thinking more agile; MET is involved in fat reduction and detoxification, as well as the synthesis of choline; THR has the function of enhancing immunity and transforming certain amino acids to achieve balance (Zhang et al., 2017). FAAs not only give BS Sufu a unique taste but also make the BS Sufu possess a certain physiological effect. The total amount of FAAs increases with the extension of BS Sufu fermentation time. In addition, through comparative studies, we found that the types and contents of various FAAs in BS Sufu are not significantly different from those of previous studies (Xu et al., 2019). However, due to the different fermentation strains used, there are certain differences in the enzyme systems during the fermentation process, which will lead to differences in the total amount of amino acids.

We identified 79 flavor constituents in BS Sufu. Ji et al., identified 35 volatile compounds in nine Sufu (Ji, 2020). Since the flavor components in Sufu are affected by raw materials, production technology, fermentation microorganisms, and environmental conditions, we believe that the effects of raw materials (black beans and soybeans) and microorganisms (three strains) make the BS Sufu rich in flavor substance diversity and higher content. Lipid oxidation gives a large number of volatile compounds or aroma precursors, which can subsequently react with amino acids derived from proteolysis to form the characteristic aroma of Sufu. The esters are the most common volatile compounds detected in BS Sufu. There are 31 kinds of ester, and their content increased with the extension of fermentation time. Esters are mainly synthesized by two pathways. One is the reaction of acyl-CoA with the corresponding higher alcohols under the action of ester synthase, and the other is the formation of organic acids and higher alcohols through esterification, but the synthesis speed of this pathway is very slow. Besides, acetyl-CoA is the precursor for the synthesis of fatty substances. Therefore, there is a certain competitive relationship between ester metabolism and lipid metabolism; but during the fermentation of BS Sufu, the abundance of carbohydrate metabolism is much higher than that of lipid metabolism (Figure 5). So, acetyl-CoA is mainly used for ester metabolism. Studies have also shown that hexanal can generate hexanol under the action of alcohol dehydrogenase, hexanol can generate hexyl acetate, and hexanal can be oxidized to hexanoic acid and can further generate esters, such as methyl hexanoate (Egea et al., 2014). Esters usually have a special aroma (Moy et al., 2012). The relative content of ethyl caprylate is 8.67% with brandy flavor, being mainly used for flavoring and spices. Ethyl phenylacetate is often found in liquor with a noticeable rose fragrance and honey-like smell. Ethyl myristate has the scent of iris oil, which is generated by esterification of myristic acid and ethanol. Ethyl caproate has a strong fruity and wine aroma and has the aroma of pineapple and banana (Kai et al., 2013). In addition, some esters also have very good pharmacological effects, such as linoleic acid ethyl ester, which has the same effect as linoleic acid in lowering cholesterol and blood lipid and can prevent or reduce atherosclerosis. Moreover, the side effect of ethyl linoleate is much lower than that of linoleic acid. BS_60 sample is rich in long-chain esters such as ethyl stearate, methyl palmitate, and ethyl linoleate. These esters improve the flavor of BS Sufu as well as health benefits. There are nine kinds of alcohols detected in BS Sufu. After 60 days of fermentation, the relative content of alcohols is significantly reduced, which may be the result of esterification or oxidation of alcohols. Alcohols often have botanical, aromatic, and earthy odors. Although their thresholds are high, they can form esters with organic acids to facilitate the formation of Sufu flavor (Wu et al., 2009). Several kinds of acids, aldehydes, and ketones were detected in BS Sufu. The aldehydes possess aromatic characteristics such as fresh fragrance, fruity and nutty fragrance, and low flavor threshold (Qi et al., 2012). Ketones are generally formed by fat degradation and oxidation reactions (Jonsdottir et al., 2008). The other volatile components detected in BS Sufu are mainly furans and pyrazines. Among them, 2-pentylfuran is mainly derived from the oxidation of linoleic acid or 2,4-decadienal, with a low threshold value and has a bean aroma (Lu et al., 2014).

The bacterial diversity of BS Sufu is higher than that of fungi. The composition of dominant bacteria in BS Sufu at different fermentation stages has a certain similarity, yet the composition of dominant fungi is quite different. *Enterococcus* and *Enterobacter* are present in the whole fermentation process, and their abundance is relatively high. *Enterococcus* with higher abundance was also found in Jiajiang Sufu (Wan H. F. et al., 2020). Similarly, *Enterobacter* and *Enterococcus* are widely present in various fermented foods, such as soy sauce, fruit juice, and tea. The abundance of the two genera in BS Sufu is relatively high, but we have measured the total number of coliforms, and the result is <3.0 MPN/g, which is lower than the national standard, indicating that the Sufu products are safe. Previous reports have shown that *Lactococcus*, *Acinetobacter*, *Tetragenococcus*, *Pseudomonas*, and *Lactobacillus* are the dominant bacteria and crucial contributors during the production of fermented foods, such as cheese, Sufu, soy sauce, liquor, and tea (Tanaka et al., 2012; Liu and Qiao, 2019). Similarly, *Lactobacillus*, *Acinetobacter*, and *Pseudomonas* were detected in BS Sufu. *Acinetobacter* and *Chryseobacterium* are abundantly enriched in BS_Y, but the amounts are significantly reduced in post-fermentation. *Acinetobacter* is also abundant in other fermented foods (Li et al., 2017; Huang et al., 2018). Previous studies have indicated that *Acinetobacter* is well known for its capacity to secrete esterolytic enzymes and is positively related to flavor compounds (Hassan et al., 2010), particularly esters.

Lefse difference analysis showed that *Lactobacillus* and *Streptococcus* were enriched in BS_M samples. *Lactobacillus* was often used in cheese fermentation (Zhang X. X. et al., 2020) and existed in other fermented products (Li et al., 2020). *Streptococcus* had a strong ability to metabolize carbohydrates (Gefei et al., 2020). The genus is enriched in the pre-fermentation stage, which may be related to carbohydrates and protein metabolism. The *Pantoea* is higher during the pre-fermentation period, and 43% of *Pantoea* is distributed in the salted pehtze (Figure 1). *Pantoea* can produce volatile compounds such as *p*-hydroxyphenylethyl alcohol, methyl 4-hydroxybenzeneacetate, and certain antibacterial properties (Mingxi et al., 2012).

Compared with bacteria, the fungal community composition is quite different. The main genera in the pre-fermentation stage are the inoculated *Rhizopus* and *Mucor*. Inoculated strain requires the accumulation of a large amount of protease and other enzymes for fermentation (Guiliang et al., 2020). *Rhizopus* and *Mucor* can secrete a large amount of protease to decompose large molecular proteins. In BS-Y, *Rhizopus* rose to 93%, which indicated that *Rhizopus* has a strong resistance to high salt environments. In the post-fermentation stage, *Monascus* is the dominant genus because *Monascus* pigment is added. *Millerozyma* is a yeast that can degrade bio-amine amines and has a certain heat resistance. It is often found in fermented wines (Jiangang et al., 2018) and also used to degrade bio-amines purposefully during the fermentation of soy sauce (Cheng et al., 2019). *Wallemia* is a salt-tolerant fungus with metabolites that have an antibacterial activity, and its fermentation products are mainly pyrrolidones and sesquiterpenoids.

*Kurthia* has a significantly positive correlation with almost all amino acids (*P* < 0.001). Current research shows that *Kurthia* can consume glucose to produce lactic acid and can produce ALA, GLU, and GLY through autotrophic pathways (Fang et al., 2015). In addition, *Staphylococcus* and Enterobacteriaceae also showed a significantly positive correlation with all amino acids, which indicated their important roles in the production and transformation of amino acids in Sufu fermentation. Enterobacteriaceae comprises various genera at the taxonomic level, appearing dominantly in fermented foods, such as doenjang, Sufu, and soy sauce, which is essential for the production of EAAs and volatile compounds (Kim et al., 2009; Devanthi et al., 2018). All amino acids have a clearly positive correlation with *Monascus* (Figure 3), which proves that *Monascus* not only provides dyes in the post-fermentation stage but also plays a prominent role during the formation of amino acids. *Mucor* and *Rhizopus* are inoculated for pre-fermentation of BS Sufu; the enzymes produced by the two molds can act directly on protein of materials involved in the production of amino acids (Zhang Y. T. et al., 2020). In addition, *Bacillus* has obviously a positive correlation with organic acids and aldehyde compounds (Figure 3). *Bacillus* can produce highly active extracellular enzymes, such as protease, amylase, and glucanase. These complex enzyme systems provide a key impetus for the formation of primary and secondary metabolites. So *Bacillus* can produce unique characteristic flavors during fermentation process, such as spicy, sweet, honey, acetic, aromatic, ethereal, and creamy (Quan et al., 2005). Therefore, *Bacillus* played a role in the production of volatile compounds during fermentation of BS Sufu. *Stenotrophomonas* can use nitrogen sources to produce peroxidase and lignolytic enzymes during growth, which can decompose macromolecular substances such as cellulose (Olajuyigbe et al., 2018). The microorganisms that metabolize aromatic amino acids and form ester flavors in the post-fermentation stage mainly include *Staphylococcus*, *Enterococcus*, *Lactobacillus*, and *Pseudomonas*. *Monascus* also has a certain correlation with amino acids, esters, alcohols, etc., while *Rhizopus* and *Apiotrichum* have a certain positive correlation with acids and ketone compounds.

Some research indicated that the number of genes in glycolysis/gluconeogenesis pathway is the most abundant in fermented foods, followed by citric acid cycle and oxidative phosphorylation (Procopio et al., 2013). In addition, carbohydrate and amino acid metabolism accounted for the highest proportion, followed by lipid and other metabolism. It proved that *Enterococcus*, *Enterobacter*, and other microorganisms with high abundance participate in the production of metabolites through different pathways. Although ester compounds have the highest correlation with *Enterococcus*, the flavor formation of BS Sufu is the result of joint action of all microorganisms in the fermentation system.

## Conclusion

In summary, we examined the microbial community structure and flavor compounds of BS Sufu at different fermentation stages by coupling HTS and GC-MS. The microbial community and metabolites in BS Sufu at different fermentation stages are significantly different. The correlation between dominant microorganism and metabolites is extremely complex, which contributes to the taste and flavor of BS Sufu. Our work provides insights into the role of microbial communities in the production of mixed soybean Sufu, and this information is important to develop a new type of Sufu.

## Data Availability Statement

The datasets (raw sequences) generated and analyzed during this study has been submitted to NCBI Sequence Read Archive (SRA) repository with accession number PRJNA693985 and can be access through following links: http://www.ncbi.nlm.nih.gov/sra/PRJNA693985.

## Author Contributions

DY and LX designed the experiments, performed most of the experiments, and wrote the manuscript. MW and XW performed the statistical analysis. KW and ZL provided valuable advice on experiments design and data analysis tools. DZ was the coordinator and guarantor, overseeing all aspects of this study. All authors read and approved the final manuscript.

## Conflict of Interest

The authors declare that the research was conducted in the absence of any commercial or financial relationships that could be construed as a potential conflict of interest.

## Supplementary Material

Supplementary Figure 1 The schematic diagram of BS Sufu production process.

Supplementary Figure 2 The microbial communities of BS Sufu were analyzed using the Lda effect size algorithm to determine the optimal characteristic taxa and rank them according to the effect size. Lda scores identified the size of differentiation; the score threshold was 4.0. Bacteria: **(A,C)**; Fungi: **(B,D)**.
