# Supplementary figures and images for: Microbial Community Succession and Metabolite Changes During Fermentation of BS Sufu, the Fermented Black Soybean Curd by Rhizopus microsporus, Rhizopus oryzae, and Actinomucor elegans
Source: Front Microbiol. 2021 Jun 25;12:665826. doi: 10.3389/fmicb.2021.665826 (PMC8267895; doi:10.3389/fmicb.2021.665826)

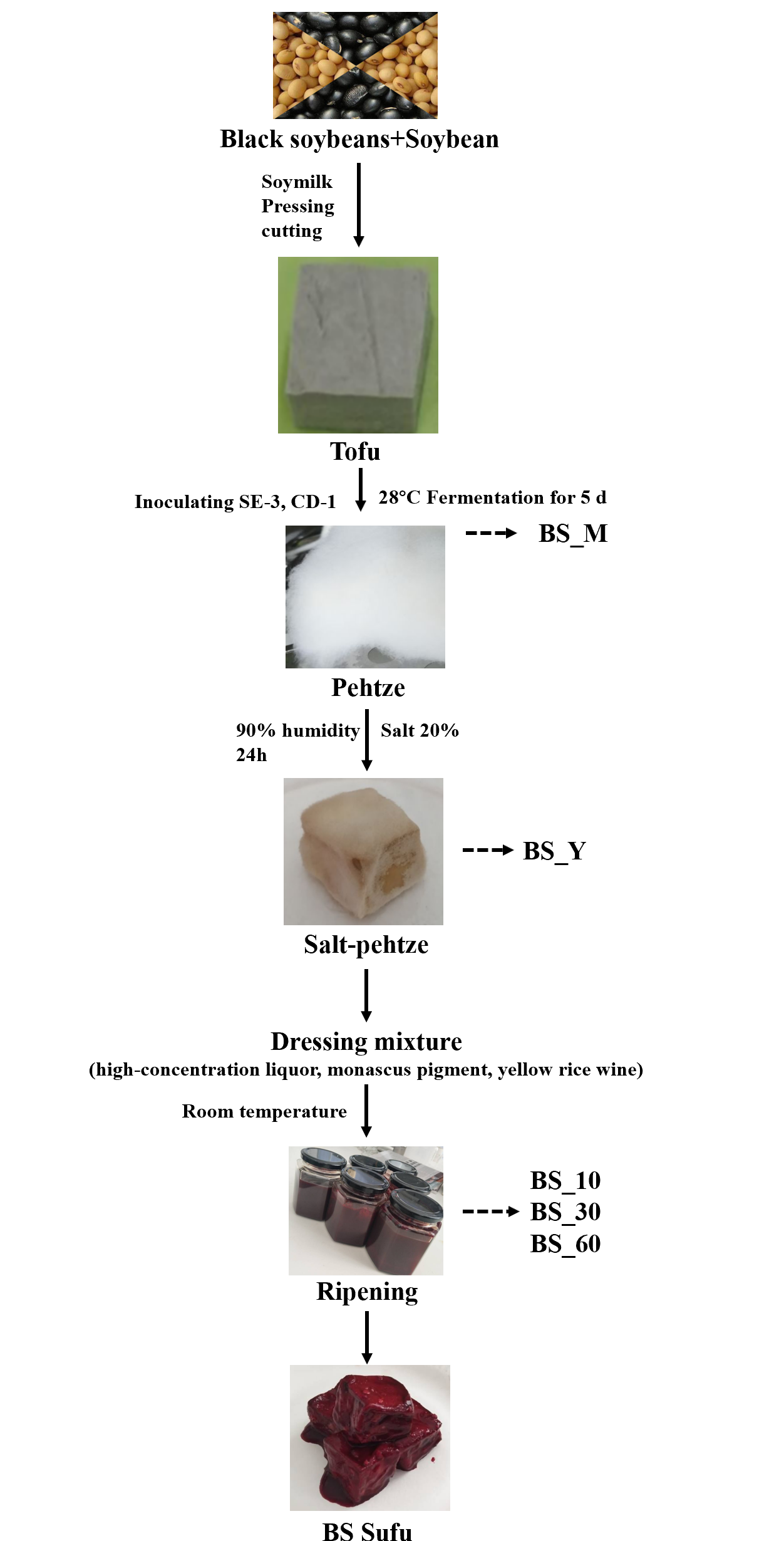

Supplement: Supplementary Figure 1 — The schematic diagram of BS Sufu production process. [file Image_1.TIF]

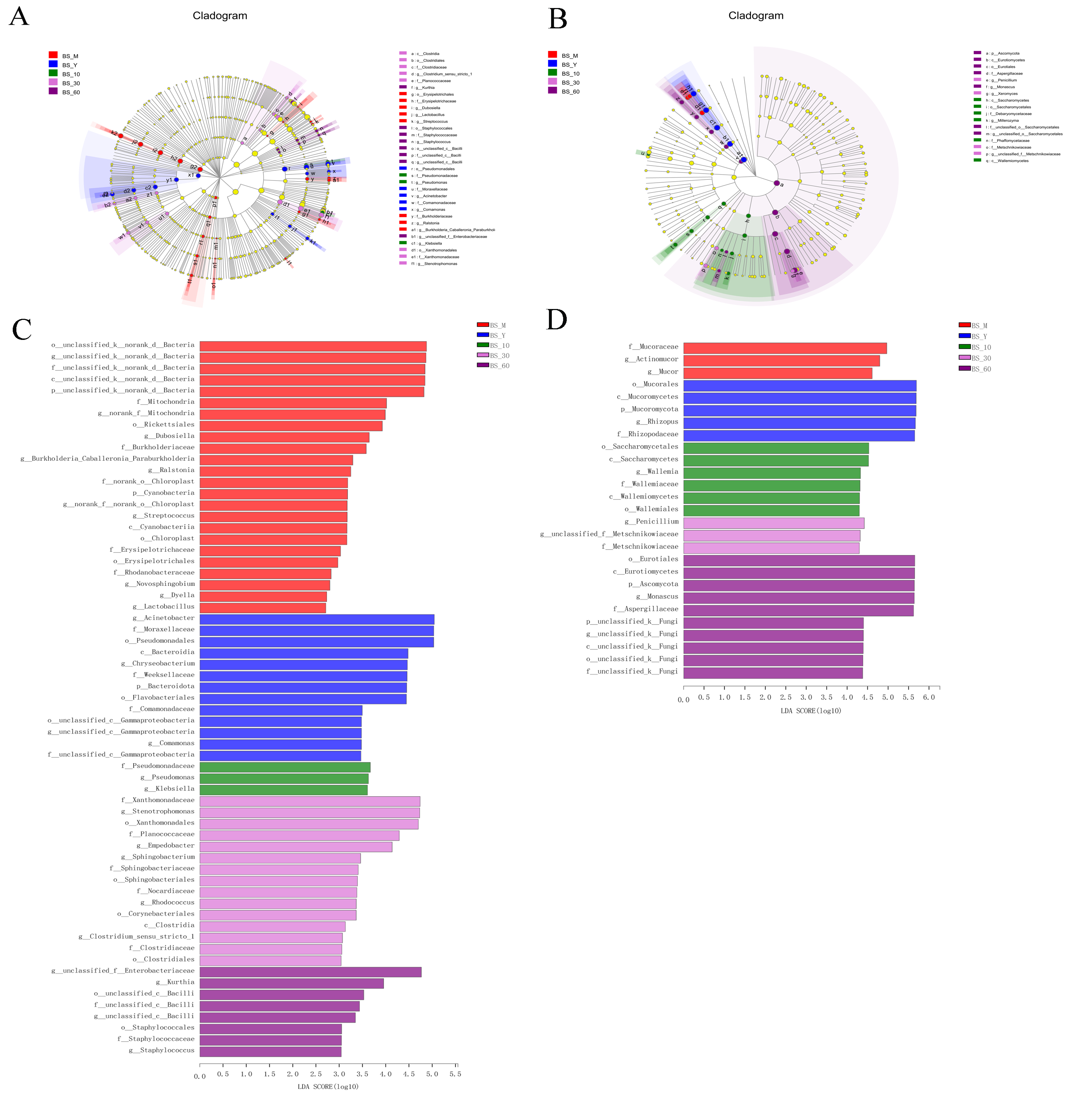

Supplement: Supplementary Figure 2 — The microbial communities of BS Sufu were analyzed using the Lda effect size algorithm to determine the optimal characteristic taxa and rank them according to the effect size. Lda scores identified the size of differentiation; the score threshold was 4.0. Bacteria: (A,C); Fungi: (B,D). [file Image_2.TIF]
